# How much biology is in the product? Role and relevance of biological evolution and function for bio-inspired design

**DOI:** 10.1007/s12064-022-00367-9

**Published:** 2022-03-28

**Authors:** Anita Roth-Nebelsick

**Affiliations:** Department of Palaeontology, State Museum of Natural History Stuttgart, Stuttgart, Germany

**Keywords:** Bioinspiration, Biomimetics, Biomimicry, Evolution, Adaptation, Function

## Abstract

Bio-inspired design (BID) means the concept of transferring functional principles from biology to technology. The core idea driving BID-related work is that evolution has shaped functional attributes, which are termed “adaptations” in biology, to a high functional performance by relentless selective pressure. For current methods and tools, such as data bases, it is implicitly supposed that the considered biological models are adaptations and their functions already clarified. Often, however, the identification of adaptations and their functional features is a difficult task which is not yet accomplished for numerous biological structures, as happens to be the case also for various organismic features from which successful BID developments were derived. This appears to question the relevance of the much stressed importance of evolution for BID. While it is obviously possible to derive an attractive technical principle from an observed biological effect without knowing its original functionality, this kind of BID (“analog BID”) has no further ties to biology. In contrast, a BID based on an adaptation and its function (“homolog BID”) is deeply embedded in biology. It is suggested that a serious and honest clarification of the functional background of a biological structure is an essential first step in devising a BID project, to recognize possible problems and pitfalls as well as to evaluate the need for further biological analysis.

## Bio-inspired engineering: terms and ideas

Bio-inspired design (BID), the concept to apply functional principles from biology to solve technical problems, has matured during the last decades into a fruitful interdisciplinary field of research with a high attractiveness to science and the wider public. This is illustrated by a steadily increasing number of publications dealing with the subject of transferring biological functionality to technical applications (Lenau et al. 2018; Lepora et al. 2013). In addition, architects and designers are drawing ideas and concepts from living nature. The attractiveness of bio-derived technical solutions has several reasons. Bio-inspired approaches require a high degree of interdisciplinarity which makes this field of work so fascinating and rewarding (Full et al. 2015). In addition, high expectations are placed upon bio-inspired technical solutions, particularly with respect to sustainability (Speck et al. 2017). These aspects combine to an attractive field which is also appealing to the wider public, all the more because “nature” and “technology” are widely perceived as opposites. A vast number of contributions were published so far dealing with quite different concepts and ideas emerging for the workflow from biology to the finally desired technical transfer. Various terms are in use for labeling approaches to practice “Bio-inspired Design” or “Biomimetics” (Hashemi Farzaneh 2020; Helms et al. 2009; Lenau et al. 2018; Speck et al. 2017; Speck and Speck 2019). For the sake of simplicity, the term BID will be applied throughout this contribution to denote practices which refer to biological structures as “concept generators” for technical innovations (Speck et al. 2008).

Although seemingly futile in view of the obvious success story of BID and its popularity, the question is: why should organisms show “superior” or “smart” functional features which can promote technical progress (or are expected to do so)? The usual explanation is, because of biological evolution, or—to be more specific—Darwin’s theory of natural selection. Typically, the anticipation is that the long-term process of evolution is only survived by those organisms which show superior traits “optimized” by selective pressure whereas individuals with inferior performance are weeded out (Wolff et al. 2017). “Trait” means all kinds of attributes of organisms, from structural characters to behavior. After millions of years of evolution, only organisms highly adapted to their environment should, therefore, exist, thanks to their highly functional traits shaped by natural selection. Therefore, the core idea of BID is biological evolution as the “producer” of traits showing novel functionality and “superior” performance formed over extended time periods by relentless pressure from the environment. From the perspective of BID, biological evolution is a natural long-term design experiment from which all sorts of technically interesting functional principles and materials can be identified (Benyus 2002; Bhushan 2009; Liu et al. 2010; Poppinga et al. 2018; Speck and Speck 2019; Wegst et al. 2015).

On the basis of this core idea, BID is tacitly supposed to refer to those approaches which are based on a clear association of the pursued technical applications and the original biological functions (Fratzl 2007; ISO 2015; Speck et al. 2017). During the last years, a number of concepts and strategies were suggested to formalize and promote BID. The heterogeneity of terminology and approaches have prompted a demand for clarifying concepts and definitions to arrive at a better structuring of bio-inspired work (Chirazi et al. 2019; Drack et al. 2017; Graeff et al. 2020; Speck et al. 2017; Wolff et al. 2017, Broeckhoven and du Plessis 2022). One critical step for the biomimetic work flow is the “abstraction from biology”, meaning the identification of the essential functional principles realized by a biological trait and to “separate” these from the biological context (Beismann et al. 2012; ISO 2015; Speck et al. 2008). Naturally, abstraction is supposed to be based on the understanding of the biological function of the trait, and how that trait works to fulfill its functional destination (Fratzl 2007).

To pinpoint the biological function of a trait appears, therefore, to be the fundamental first step when a “biological solution” is searched for a technical problem. In the BID literature, problems are usually addressed which are beyond the core of biological trait-function relationships and their evolution. Considered are, for example, the importance of precise identification and analysis of working principles (Wolff et al. 2017), the quite complex relationships between biological functions and BID-derived technical applications (Speck et al. 2017), methods and strategies for assessing and/or improving BID-related “solution-finding” (Helms et al. 2009) or “biomimetic promises” of sustainability (Antony et al. 2016; Mead and Jeanrenaud 2017). During the last years quite a few approaches and methods, or “tools”, were devised to support and structure the biomimetic workflow (Wanieck et al. 2017). A substantial part of these tools is represented by biomimetic data bases which are deemed promising for an efficient identification of suitable biological models to solve technical problems (Bae and Lee 2019; Fayemi et al. 2017; Goel et al. 2014; Lenau et al. 2018; Vincent et al. 2006; Wanieck et al. 2017). In addition, strategies to facilitate and increase commercial success of innovations based on BID are addressed (Chirazi et al. 2019).

These approaches and methods focus on “post-biological-function” aspects, meaning that basic knowledge on the function(s) of a biological trait is largely taken for granted (because it is considered to be already solved) in BID-related work, and therefore, does not merit further consideration. In addition, the assumption that biological structures are shaped by evolution to a “high-performance” state appears to be mostly accepted, and is only occasionally discussed (Fish and Beneski 2014). Moreover, it also appears that BID data bases may be attractive for making BID-related work less dependent on biologists and/or biological expertise (Graeff et al. 2019, 2020).

The present contribution takes a step back, to the core idea of BID. How valid is the BID perspective on biological traits, their functions, and their evolution? In fact, the topic of “function” of a biological trait, its identification and the underlying selective forces are often anything else than trivial, and sparked decade-long major debates among biologists together with substantial methodological advance. Should this be of concern for BID, as these aspects touch its central assumptions and justifications? Does it matter? It will be attempted in this contribution to (1) discuss the core idea of BID by briefly describing some current fundamental views in biology on the interrelationships between biological functions and evolution, (2) evaluate these interrelationships as a key element for devising BID concepts, and (3) discuss how essential the biological context of a function is for successful BID.

## BID as an adaptationist program

### Functions and purposes in evolution

Since the “function” of a biological structure is of central meaning for BID, it seems necessary to first clarify what “function” means in biology. Although seemingly trivial, the term “function” is in fact quite ambiguously used and its meaning and proper definition is a subject of a long-standing debate (Krohs and Kroes 2009; Wouters 2005). Consider, for example, the function of the hierarchically structured wax crystals which cover the leaves of various plants showing the Lotus® effect (meaning extreme water repellency of superhydrophobic surfaces) (Barthlott et al. 2016; Barthlott and Neinhuis 1997). What is the function of these wax crystals? To provide a superhydrophobic contact angle? To repel water vigorously? To ensure self-cleaning? And why, in the first place, should self-cleaning be beneficial for a leaf? As pointed out by Neinhuis and Barthlott (1997), self-cleaning removes pathogens, such as spores of fungi, from leaf surfaces. Is prevention of fungal infections, then, the function of these kind of wax crystals?

“Function” evokes an intentional action, a planned “means-ends relationship”, which is clearly problematic in the biological context. Evolution itself has no intention (Fish and Beneski 2014), and Darwin´s theory of natural selection made it possible to explain the seemingly “ingenious problem-solving” in living nature as driven by an unconscious process, without referring to teleology. Of course, the use of metaphors and analogies related to intentionality is widespread to explain the evolution of biological traits and was also employed by Darwin himself (Pramling 2009; Young 1985), simply because this is an efficient and pedagogically useful way to explain things which would be very complicated to explain otherwise. This is undoubtedly true, but carries the problem of intentional thinking “sneaking” into the subject of biological functionality. How can “function” be defined and understood for biological traits?

**Fig. 1:**
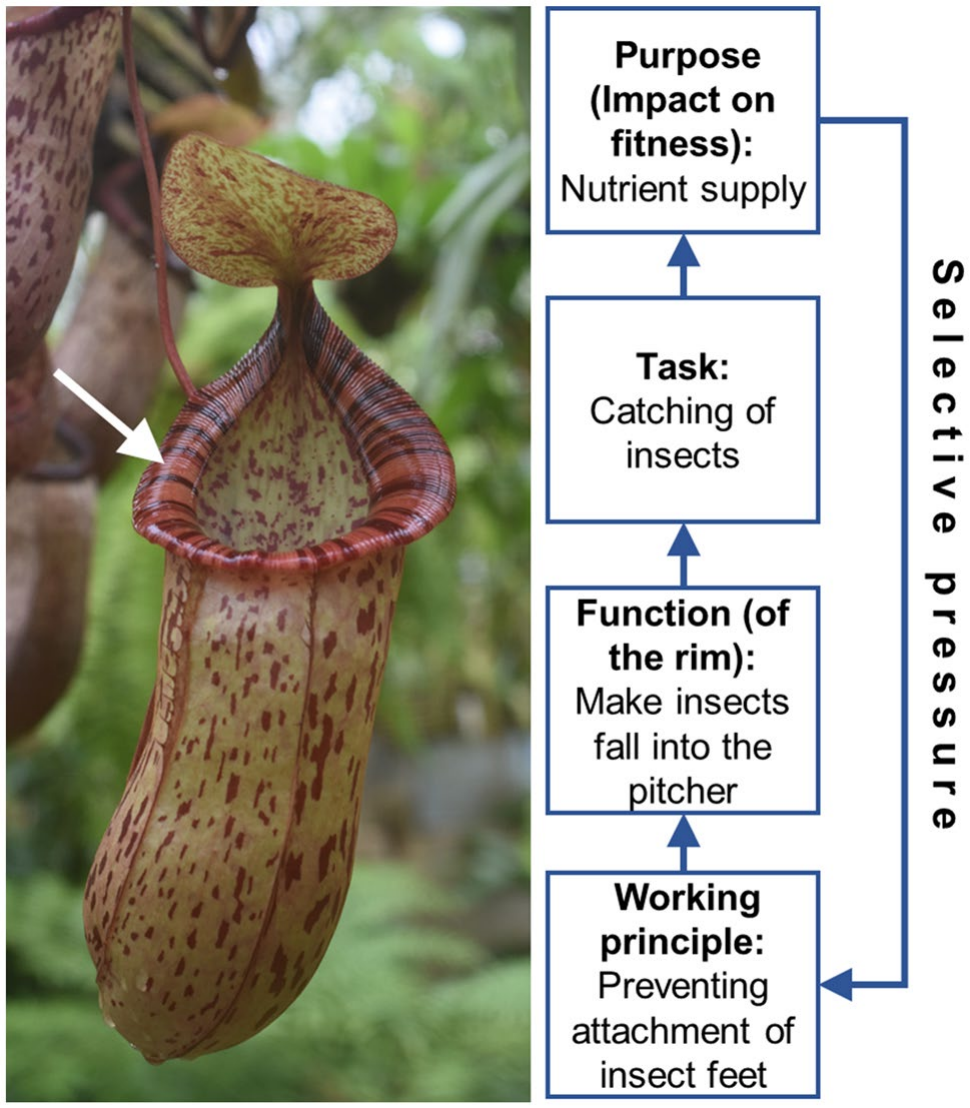
The “functional catena” of a biological trait, illustrated here by using the example of a pitcher plant (*Nepenthes ventricosa* x *Nepenthes spectabilis* hybrid, Botanical Garden of the Wilhelma, Stuttgart, Germany). The pitcher is an insect trap, build by special leaves and has various functional elements. Here, the pitcher rim is considered (white arrow). The function of the rim is to make insects slip so that they fall into the pitcher. The working principle employs various surface effects which prevent insect feet from attaching to the rim surface. The task is catching insects for the ultimate purpose of anorganic nutrient supply (nitrogen and phosphorus) which enhances fitness and, therefore, reproductive success. The functional structures are, therefore, steadily “trimmed” to a high-performance level by selection, via differential reproductive success

To provide an unambiguous definition, Drack et al. (2017) introduced a concept borrowed from engineering design (Pahl and Beitz 2013). Here, “function” appears as a component of a catena, ranging from “Working principle” to “Task”. While the task of a device can be described with the question “what for?”, the working principle is the physical process put to work by the device. The element “function” is situated between task and working principle and is described by the “action” which utilizes the working principle to fulfill the task. This function concept from engineering is suitable to describe also biological function because it allows to identify hierarchical processes leading finally to evolution. Figure 1 shows as an example the representation of the functional levels of the insect trap formed by carnivorous pitcher plants.

Pitcher plants show vessel-like traps—which are specially shaped leaves—catching insects. Rim and internal walls of the traps hamper attachment of insect feet, making (1) the insects fall into the trap, and (2) prevent caught insects from escaping the trap. For the sake of simplicity, the example will focus on one component of the pitcher trap, the rim. The task of the rim may be formulated as “let insects fall into the pitcher”. The way how insects are made to fall into the pitcher is by slipping. The function can, therefore, be described as “make insects slip” so that they—ultimately—fall into the pitcher, task fulfilled. The function, the slipping of insects, is based on the working principle, which is represented by physical effects of the rim surface hindering insect feet to attach to it (Bauer and Federle 2009; Gorb and Gorb 2011; Gorb et al. 2014; Scholz et al. 2010).

Now, we can add another level on top of the task, by asking further questions: what is the task of the rim good for? Why should insects slip and fall into the pitcher? This sounds trivial: to supply the digestive system with prey, of course. But what use do have pitcher plants for caught insects? Pitcher plants do photosynthesis, they do not “eat”. As in all carnivorous plants, pitcher plants utilize the inorganic nutrients of their prey, particularly nitrogen and phosphorus, and not their caloric content. Supply of nutrients enhances productivity in a plant and will, therefore, contribute to fitness. Here comes the link to evolution: the “purpose” of the whole process—to employ this intentional expression—is to enhance fitness by exploiting additional sources for nutrients. The better the function of the rim is fulfilled, the higher is the success of nutrient capture, meaning that reproductive success of individual plants will increase with the ability of the rim structures to realize the working principle. These structures are, therefore, steadily “trimmed” to a high-performance level via differential reproductive success. In this way, a “loop” of selective pressure is closed between the ultimate purpose of the effect and the functional trait structure(s) (Fig. 1).

### Adaptation and plasticity

Traits whose “purpose” is to enhance fitness are termed adaptations (Gardner 2017). The rim of the pitcher trap is, therefore, an adaptation, as are all other elements of the trap with their specific functions involved in prey catching, digestion and nutrient absorption. If a trait is an adaptation, it can, first, be expected to be shaped by evolution to a high (or at least “sufficient”) performance. Second, only in the case of an adaptation, there is a function, with an underlying working principle realizing that function. Otherwise, there is only an “effect” to be observed, without biological relevance. A general definition of “adaptation” is provided by Vermeij (1996): “Adaptation is a heritable attribute of an entity that confers advantages in survival and reproduction of that entity in a given environment”. This definition includes two important aspects: first, that the considered trait is heritable, and second, that the fitness benefit is environment-specific. Moreover, adaptations represent products as well as drivers of natural selection, because “adaptation” denotes the trait as well as the process of natural selection which shaped it (Vermeij 1996).

Adaptations are, therefore, assumed to be the product of evolution. It is necessary at this point to separate phenotypic plasticity from adaptations. In other—non-biological—contexts, “adaptation” means responses and/or adjustments of a system to various external cues, triggers and conditions, and “adaptive” means its ability to do so. For example, in architecture, the term "adaptive façade” describes exterior elements of buildings which are able to change their shape, for example in response to insolation (Romano et al. 2018; Schleicher et al. 2011). For organisms, the ability to adapt body structure and also physiological processes to environment is termed plasticity and represents an aspect of biology which is also attractive for BID (Gebeshuber and Drack 2008). As an example, tree trunks can adopt different shapes and heights, according to the topology of the terrain (such as slopes), availability of water and nutrients, wind load and other factors (Burgert and Jungnikl 2004). If clones of an individual tree were planted in different environments, each clone would develop an individual shape. Plasticity can often be considered as an adaptive trait itself which is under genetic control (Chitwood and Sinha 2016; Dudley 2004). Leaves in the upper and exposed regions of a tree canopy (sun leaves), for example, are often smaller and thicker than leaves which are situated closer to the ground and in the shade (shade leaves) (Terashima et al. 2001). This plasticity of leaves within one individual tree allows to fine-tune the leaf structure according to the immediate environment, for example to develop more layers of assimilating cells at sun-exposed sites of the tree, and is considered to be an adaptation.

### The adaptationist program

All this sounds as if adaptation is exactly what is being sought in BID. However, despite its seemingly obvious role as a key term in evolutionary biology, the concept of adaptation has a quite checkered history. In fact, there were widespread and fundamental discontents and debates on adaptation during the last decades. These were so severe, that—at a certain time—some avoided the word itself. In the foreword to their book “Adaptation”, Rose and Lauder (1996) recalled: “One of us attended a seminar in the early 1980s at which the speaker announced that he would not use the word adaptation in his talk. Rather, to avoid controversy and association with the negative implications of adaptionism, he would use the word ´banana´ whenever he meant adaptation.”

How could that be, given the fact that there are obviously countless numbers of traits which can be hardly explained otherwise than being adaptations? These (partially heated) debates were sparked by an article by Gould and Lewontin (1979) who criticized the “adaptationist program”. One point of critique was that “adaptationists” would assume a priori that each trait has a function, taking not into consideration the possibility of other reasons for the existence of a trait, and, moreover, accepting “just so stories” for functional explanations. Another point of critique raised in Gould and Lewontin (1979) was the “atomization” of an organism into single traits and to attempt to explain isolated traits as adaptations without considering the total performance of the entire organism with respect to fitness (this touches also the problem of trade-offs, a topic of high relevance in BID (Vincent 2017)).

The impact of the contribution of Gould and Lewontin (1979) was huge, with thousands of articles being published in its wake, until today. For a longer time now, it is no longer necessary to say “banana” instead of “adaptation” to prevent unrest but high(er) standards for proving that an adaptation is an adaptation were demanded as one consequence. Moreover, new results and methods have accumulated since then which allow for a “sharper” and sound separation of adaptive and non-adaptive drivers in trait evolution and also to unambiguously identify function and task of traits. In this respect, progress in phylogenetics, the field dealing with evolutionary history of species and how species are related, are highly relevant. All these widespread and fundamental discontents and debates on adaptation and biological function during the last decades should be relevant for BID because—obviously—BID represents a kind of an “adaptationist program”.

## Identifying adaptations

### Is there work for the working principle?

Recapitulating the “functional catena” of a biological trait, there is the working principle, employed by the function which fulfills a task. This task enhances fitness as the ultimate purpose of that trait which can, therefore, be classified as an adaptation, because it was shaped by evolution. BID pursues to tap the functionality of adaptations and to transfer those into technical applications. There are numerous examples which appear to suggest that biological functions or tasks, and therefore, adaptations, are rather obvious. For example, the ability of flexible elephant trunks to handle food or other objects, the snapping of the venus fly trap to catch insects, cutting and crushing of food using teeth, locomotion by flight conveyed by wings or locomotion by running on legs are quite obvious examples.

However, there are countless other cases in which the “use” of a trait is not so evident. For example, fossil organisms can obviously not be observed, and therefore, the “use” of a trait in an extinct species has to be reconstructed which can be painstaking and may not go beyond speculation. Adaptations can, however, also be far from being obvious in extant organisms, because the function cannot be observed “in action”, but is contributing “silently” to the overall performance of the organism. For example, why do glass sponges possess silica spicules which show remarkable fiber-optical properties and are able to transmit light (Sundar et al. 2003)? Sponges have no eyes, they are sessile and do not conduct photosynthesis, so why should light be of any relevance for them? In fact, there is a benefit, namely to provide light to certain algae living inside sponges. The sponges benefit from the photosynthesis products of their algae “endobionts” (Brümmer et al. 2008), and the light-transmitting fibers of glass sponges can, therefore, be considered as an adaptation.

**Fig. 2:**
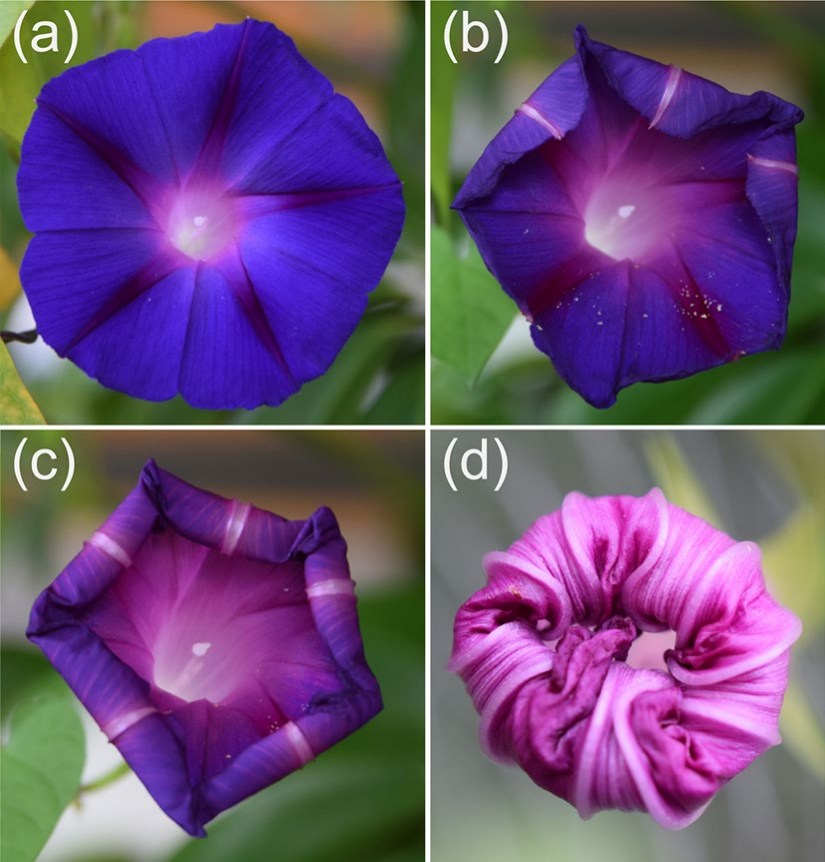
Closing of a flower of *Ipomoea purpurea*, the morning glory. Images **a**–**d** show this process. As in many species, the flower does not close passively by wilting, but by a complex process. In *I. purpurea*, the petal midribs perform differential growth, leading to curling up of the petals until the flower is closed. While opening of flowers allows pollination, the functional background of closing is more obscure. Please note that the images are not to scale, because the diameter of the flower decreases during closing. Note also that the color of the petals changes during closing

“Silent functions” can also hamper the clarification of “active and obvious” functions, because many structures represent a functional complex composed of different elements which contribute to the overall functionality, but whose single roles in that functional complex are unclear or unknown. Also in the case of obvious “activities”, however, the adaptive benefit can be enigmatic. An example is opening and closing of flowers. It is quite out of the question that the ability of flowers to open is a necessary trait to allow pollination. Flower opening can be quite spectacular and is, in fact, considered as a biological model for deployable structures (Kobayashi et al. 2003; Schleicher et al. 2015). What about, however, flower closing which occurs in many plant species? For example, species of the genus *Ipomoea* show not only an impressive unfolding of the petals but also a regular closing process of the flower, some hours after opening (Fig. 2). The closing is not brought about by mere wilting of the petals. Rather it is caused by movements of the midrib due to differential cell elongation (van Doorn and van Meeteren 2003). Why do these flowers close, or: where is the possible enhancement of fitness? It was suggested that flower closure in various plant species occurs because the pollen loses viability rapidly and flower closure would prevent pollination with less vital pollen, hence enhance fitness (Franchi et al. 2014). While data appear to support this hypothesis, it cannot yet be considered as proven (van Doorn and Kamdee 2014). Moreover, there are numerous other cases of flower closure which are obviously not due to this cause (van Doorn and Kamdee 2014).

An unambiguous identification of an adaptation requires in many cases painstaking work. The adaptive status of a trait is usually evaluated by combination of different methods and approaches, and computer simulations are frequently helpful or even required (Wolff et al. 2017). Often, it is necessary to study life style and habitat of the organism to understand performance and adaptive benefit of an assumed functional structure, meaning observations of the considered organism in its natural environment (Arnold 1983; Grun and Nebelsick 2018; Higham et al. 2019; Koehl 1996; Taylor and Thomas 2014). For example, the filter apparatus of the copepod *Eucalanus pileatus* is not a passive filter but actively deployed and moved by the animal, and revealing its efficiency requires observing the undisturbed animal (Koehl 1996). In addition, for traits with seemingly obvious functions, things can turn out to be not so clear. For example, the long neck of the giraffe is commonly explained as providing access to leaves growing at higher positions of tree canopies, thereby benefitting giraffes compared to other browsing animals which have shorter necks. Some years ago, however, this explanation was questioned on the basis of field observations on browsing behavior (Simmons and Scheepers 1996). On the basis of these observations, it was suggested that the long neck of the giraffe plays a role in male combat. Forcefulness of clubbing opponents increases with the length of the neck, and long-necked males would, therefore, dominate and benefit with respect to mating (Simmons and Scheepers 1996). Long necks in the giraffe would then not function like a crane but rather as a club.

**Fig. 3:**
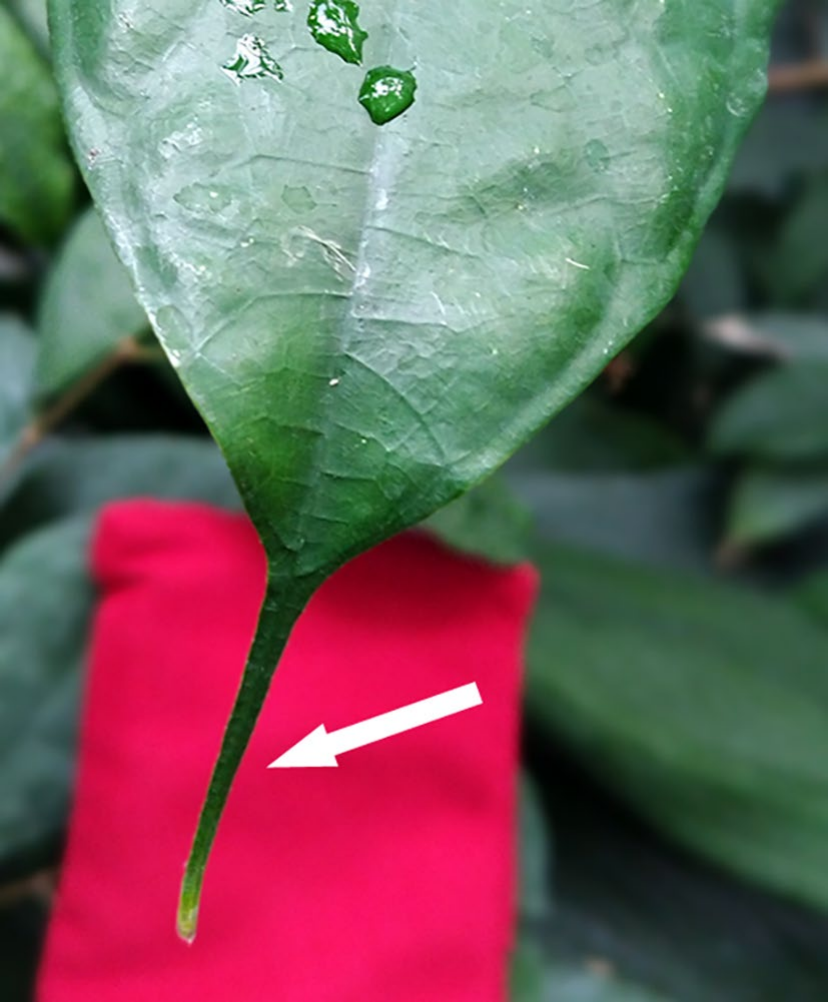
In various plant species, leaves show a distinctly elongated tip, as shown here for *Ficus sinuata* (white arrow, leaf tip before red background). According to its name, “drip tip”, this feature is often interpreted as facilitating run-off of rain water. There is, however, no unequivocal evidence for this assumed benefit

To further illustrate frequent problems with assumed adaptive benefits, the story of the “drip tip” will be explained as a simple example to some detail. A drip tip means a substantially elongated leaf tip which occurs particularly often in leaves of plants living in tropical environments (Fig. 3). For a long time, drip tips were explained as an adaptation to wet environments with the function of accelerating the shedding of rain water (Jungner 1891), thereby aiding rapid draining and drying of the leaf surface after rainfall. As task, it was suggested that rapid drying of the leaf surface would prevent other organisms from growing on it. Some experimental results did in fact support the idea that drip tips promote water-shedding (Ivey and DeSilva 2001; Lightbody 1985). Evidence for the supposed adaptive benefit of rapid water run-off provided by drip tips is, however, weak. First, no effect of drip tips on the growth of organisms settling on leaves (the assumed adaptive benefit of drip tips) could be detected so far (Burd 2007; Lücking and Bernecker-Lücking 2005; Monge-Najera and Blanco 1995). Second, the speed and efficacy of water-shedding is actually not dominated by a drip tip, but depends also heavily on various other parameters, particularly surface properties (wettability and contact angle hysteresis), surface profile and leaf angle (Holder 2007; Konrad et al. 2012; Lenz et al. 2022; Roth-Nebelsick et al. 2022). Furthermore, drop impact lead to a transient change in leaf inclination, by “pushing” the leaf downwards, affecting water-shedding off leaves during a rainfall event (Ginebra-Solanellas et al. 2020). In addition, leaves with drip tips appear to be no more frequent in humid forests than in drier forests, and any positive correlation between water repellency of leaves and humidity and/or temperature appears to be absent (Ellenberg 1985; Goldsmith et al. 2017; Holder 2007). Quite the contrary, water repellency of leaves was observed to decrease with increasing humidity (Goldsmith et al. 2017).

In summary, it can be stated that there is no real evidence for the assumed function of drip tips to promote water-shedding. Even if there would be a real physical effect which makes an elongated tip supporting drainage, this effect could be hardly—at least at the current state of things—called “working principle” because there is no proven benefit served by it, and therefore, no function identified so far (and consequently, no work for the putative working principle). This quite simple example illustrates not only the difficulties which can be encountered when attempting to identify a trait as an adaptation. It also shows that the natural environment, the habitat and the ecological niche of the organism is crucial for proving adaptations.

### Species are related: helpful and problematic for identifying adaptations

**Fig. 4:**
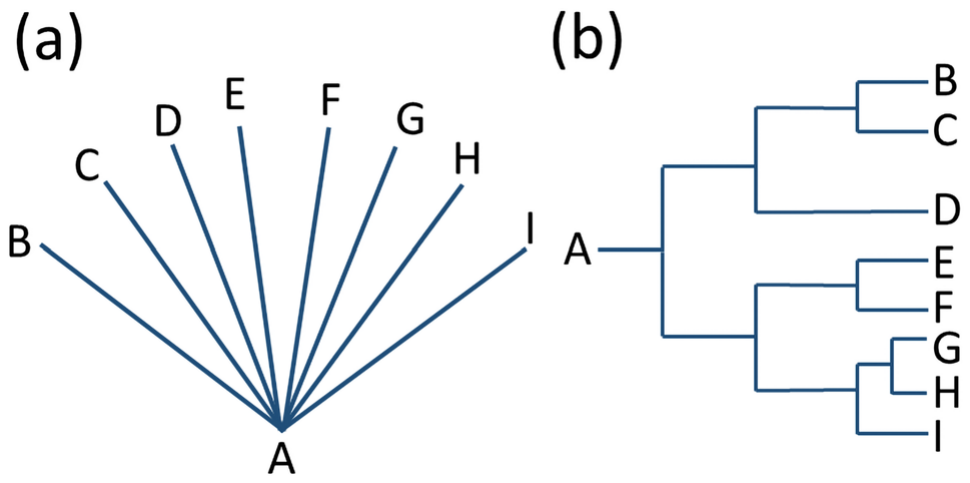
**a** When different species (B–I) evolve from a shared ancestor (A), they do not “split up at once”, which would result in the same degree of relatedness, as shown in this sketch. **b** Rather, speciation events starting from a shared ancestor occur subsequently, while the time span between speciation events also differ. The result is a “phylogenetic tree” shown in this sketch, with different degrees of relatedness between the species (B–I). When traits of a set of related species (such as B–I) are compared to find evidence for selective forces shaping adaptations, tests for simple correlations between trait data and environmental and/or life style data are, therefore, not adequate. Special methods are necessary to account statistically for the complex data structure represented by a phylogenetic tree

Often, evidence for functions served by a trait and/or adaptive “improvement” of a trait (by evolution) is sought by testing a number of species for correlations between trait data and environment or life style. For instance, in a comprehensive study, Taylor and Thomas (2014) identified various wing traits of birds as adaptations to certain soaring habits. Comparative studies have, therefore, great potential to prove adaptations. This kind of analysis, however, has to cope with an essential confounding factor, namely the circumstance that species are related. Evolution means that different species developed from a shared ancestor, in a process termed phylogenesis, and a “phylogenetic tree” describes the “family tree” of different related species (Fig. 4). When species evolve from ancestors, they do genetically not start from zero. Lineages, meaning groups of species with a shared ancestry, do also share certain features due to a shared genetic heredity. Species which are closer related will tend to be more similar to each other than species which are more distantly related (this is sometimes termed as “phylogenetic inertia”).

Similarity between species may, therefore, just be due to relatedness. For example, all members of the plant group Pinophyta, also known as conifers, have tracheids as water-conducting cells. This kind of “ancestry baggage” is termed phylogenetic constraints and has the consequence that traits shown by different species cannot be considered as independent data points (Fig. 4). The “relatedness” of species has to be taken into account when evaluating the adaptive significance of a trait, and various methods for separating both factors were devised during the last decades (Desdevises et al. 2003; Felsenstein 1985; Harvey and Pagel 1991; Münkemüller et al. 2012; Taylor and Thomas 2014). This problem will be illustrated in a biomimetic context in the following with some examples.

Insect wings are of biomimetic interest due to various properties, such as a high diversity of nanostructuring and surface effects. For example, various cicada species feature superhydrophobic wings (Watson et al. 2008). The anticipation is that superhydrophobic wing surfaces should be of adaptive benefit in a moist environment. In a recent comparative study, it was investigated whether and how wettability of different cicada species depend on their natural habitats (drier vs. wetter) (Oh et al. 2017). The data showed that wettability was more dependent on relatedness of the considered species than on their typical environment (Oh et al. 2017). The results were, therefore, not consistent with expectations based on “evolutionary improvement” of a trait-function complex.

Another example refers to the ability of fog collection. Fog harvesting is considered as a promising method to improve freshwater supply in dry areas and there are ongoing efforts to improve artificial fog collectors, including biomimetic approaches (Azad et al. 2015; Gurera and Bhushan 2019; Ju et al. 2013; Klemm et al. 2012; Shigezawa et al. 2016). Fog (and dew) are recognized as potentially important water sources for plants (Dawson 1998; Eller et al. 2013; Simonin 2009) and this motivates the search for special plant traits that might improve fog collection (Andrews et al. 2011; Azad et al. 2015; Gurera and Bhushan 2019). Expectedly, selective pressure for traits that improve fog collection should be particularly high in areas showing low precipitation but frequent fog events. A plant which appears to be promising with respect to fog collection is the pine species *Pinus canariensis* which is endemic to mountain regions in the canary islands where orographic lifting of moist winds leads quite frequently to fog formation on slopes (Fernández‐Palacios and de Nicolás 1995). In fact, growth of *P. canariensis* was found to be adversely affected at sites with lower fog frequency (Rozas et al. 2013), and a selective pressure towards efficient and effective fog harvesting may, therefore, be anticipated for *P. canariensis*. For example, it may be speculated that various characteristics of needles of *P. canariensis*, such as minute barb-like structures running along the needle, are adaptations to promote fog harvesting.

To detect possible adaptive traits for enhancement of fog collection in *P. canariensis*, a comparative study was conducted by the group of the author. This study included anatomical investigations and measurements of fog harvesting ability for the needles of a number of different pine species, including *P. canariensis*, and artificial reference objects. Performance of fog harvest was measured and expressed as interception efficiency (IE) which is the ratio between fog collection rate and surface area of the objects. When the results were compared, it turned out, first, that IE is dominated by diameter, as was expected due to the positive correlation between diameter and boundary layer thickness of an object. Second, the needles of *P. canariensis* showed no superior ability for fog harvesting, compared to other pine species. Subsequently, it was tested whether the trait “needle diameter” (together with a number of other traits) in *Pinus* shows any correlations with environmental parameters and/or depends on phylogenetic relationships. The results revealed that pine needle shape is correlated to a large degree with mean annual temperature (MAT), with both the needle width and the ratio of needle width to length decreasing with MAT (Nobis et al. 2012). The reasons why pine needles are shorter and broader in cold climate than under warmer conditions are still conjectural. Suggestions comprise freezing (Kaku and Salt 1968), snow load or growth conditions (Jankowski et al. 2017). To cut a long story short, there was no evidence for special surface traits or other adaptations conveying superior fog harvesting abilities to needles of *P. canariensis*, and their long thin shape is a general trait in needles of pine species which live in warm and frost-free habitats.

In another case, however, the anticipation that dry environments with frequent fog events promote adaptations for fog harvesting was fruitful. A certain grass species, *Stipagrostis sabulicola*, is endemic to the hyperarid Namib desert and is able to contribute substantially to the Namib biomass even during exceptionally dry years (Southgate et al. 1996). Fieldwork indeed confirmed high fog harvesting qualities of *S. sabulicola* (Ebner et al. 2011). The factors contributing to this ability are mainly the shape of the plant (a stand of stiff culms with heights up to 2 m combing out fog water quite efficiently) and its rough surface with longitudinal grooves. This surface allows for development of large drops which are then conducted quite reliably to the plant basis and the roots (Ebner et al. 2011; Roth-Nebelsick et al. 2012). Compared to other species of *Stipagrostis* which also grow in the Namib, the rough and grooved surface of *S. sabulicola*, its persistence and its height distinguishes it clearly from these other *species*. It is, therefore, reasonable to assume that various traits of *S. sabulicola* represent adaptations to fog collection and served as inspirations in a biomimetic context (Gurera and Bhushan 2019; Park and Kumar 2017; Wu et al. 2017).

The examples described so far demonstrate that identifying adaptations may be quite difficult and time-consuming, and may well lead to negative results. The question also arises whether traits are always adaptations. This question appears to be odd at first sight. Why and how should “useless” traits appear if they have no function and task, or, in other words, which are not produced by selective forces acting upon them? There are various reasons. Some are caused by the arrangement of the genetic system. For example, one gene can be involved in the expression of more than one trait (pleiotropy) which leads to the result that mutations in that gene will also affect more than one trait. Selection for variants of one of these traits will automatically work also on the other coupled traits, as a side-effect. Another example is due to the circumstance that chromosomes comprise many genes: these are then “linked” (like pearls on a string). Suppose that the genes A and B are located on the same chromosome. Variants of these linked genes will then occur together, for example, variant A_1_ will be linked with variant B_1_ and variant A_2_ with variant B_2_. Selection for A_1_ will then automatically increase the frequency of B_1_, as a side effect which is called hitchhiking. An example for obtaining a number of trait variants by selecting for one single trait is the “domestication syndrome”. Here, selection for one single trait, namely tameness, leads to a whole suite of differences between domesticated animals and their wild ancestors (Kukekova et al. 2018; Wilkins et al. 2014).

There may be also reasons for the appearance of traits or trait changes other than selection or mutation (and which are then not heritable), as illustrated by the following rather curious example. A large number of people worldwide are infected with the protozoan parasite *Toxoplasma gondii* which is spread by cats (Dubey and Jones 2008). *T. gondii* also infects other animals, such as rodents, and is capable of altering the behavior of its hosts towards more risk-taking. This is very likely an adaptation of *T. gondii* which serves to increase the probability of the host for becoming a prey of cats in which *T. gondii* finally reproduces sexually (Dubey and Jones 2008). There is evidence that *T. gondii* also influences the behavior of its human hosts, and a recent study addresses the possibility that human infection with *T. gondii* promotes the inclination of infected persons to the risky business of entrepreneurship (Johnson et al. 2018). In this case, this inclination would not be a personal trait but rather the consequence of an infection with *T. gondii*.

### A heretical question: is the biological function really important for a successful BID?

Naturally, the question arises whether dealing with all these kinds of problems and efforts described in the preceding chapters are really necessary for BID. After all, BID aims at finding innovative technical solutions, and one may adopt the viewpoint that biological complications of the considered trait are not the concern of a biomimetic project. One could go a step further and ask how relevant the biological function of a trait really is for a technical development which is based on that trait. Not rarely, a successful biomimetic design was derived from traits whose biological functions are still unknown or under debate. To illustrate this, some BID examples will be discussed.

**Fig. 5:**
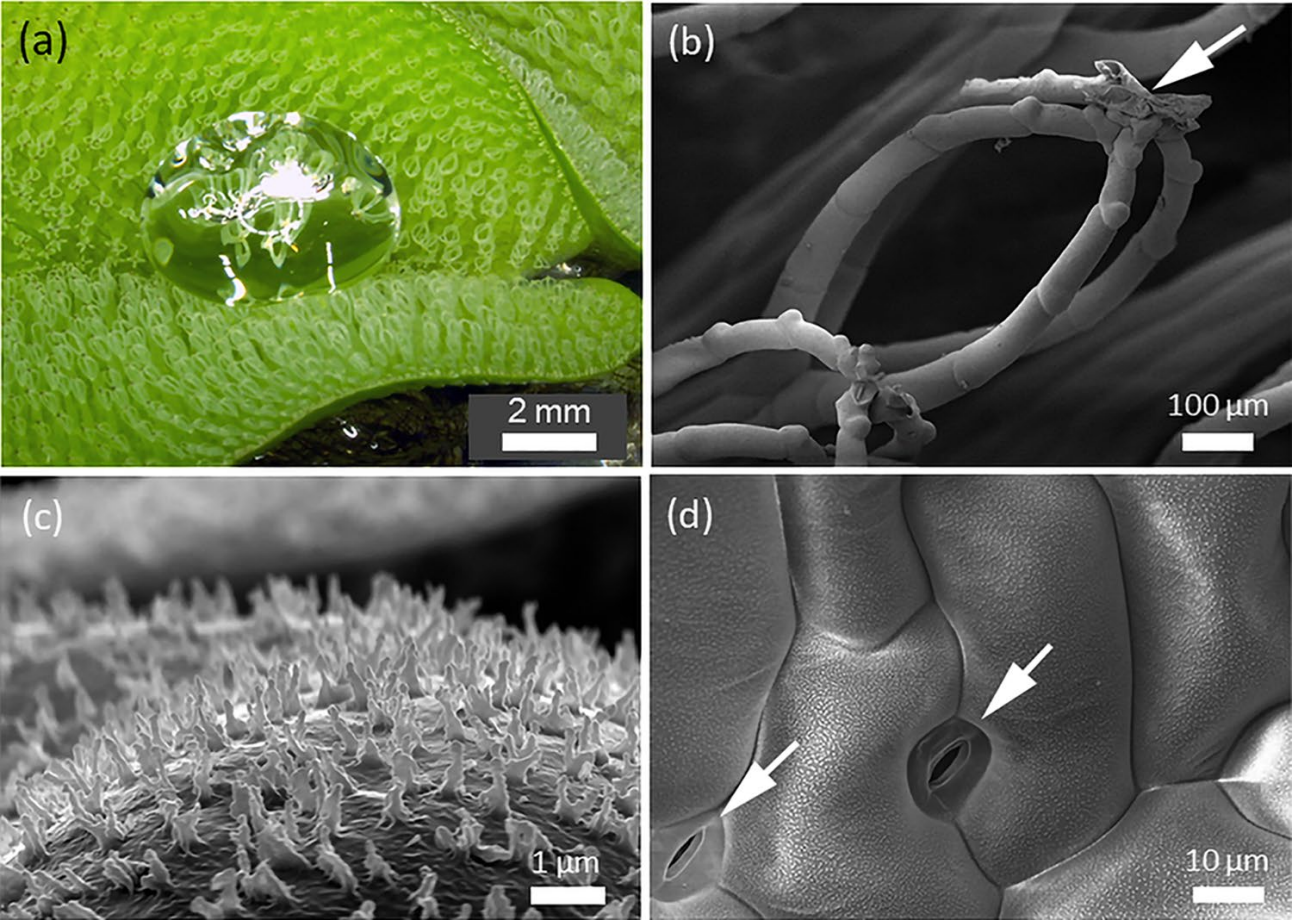
Surface structures of the floating water fern *Salvinia molesta*. **a** The upper leaf surface of *S. molesta* is covered with plant hairs (trichomes). The surface is superhydrophobic and is also able to keep a persistent air layer when immersed in water. **b** The trichomes of *S. molesta* are multicellular and the upper part shows a complex “egg-beater-like” appearance. The very tip of the “egg-beaters” are topped with a hydrophilic structure (white arrow). The rest of the trichome surface as well as the entire upper leaf surface are superhydrophobic. **c** Superhydrophobicity is caused by special wax crystals which cover the upper leaf surface including the trichomes. The image shows wax crystals on a trichome. **d** Wax crystals covering the upper leaf surface. The image also shows two stomata (gas-exchange pores, white arrows)

In *Salvinia*, a group of species of floating ferns, the upper leaf side is covered with hydrophobic hairs (= trichomes) (Fig. 5a). These hairs are able to keep a persistent air layer when the leaf is immersed in water (Barthlott et al. 2010). Superhydrophobic surfaces are usually enveloped by an air layer when immersed in water, but the layer is not stable and disappears quite rapidly. In contrast, the upper leaf surface of *Salvinia* species, such as, for example, *Salvinia molesta*, is able to keep the air layer under water for days (Ditsche et al. 2015). This phenomenon was termed “Salvinia Effect” (Koch et al. 2009). *S. molesta* shows a quite spectacular type of surface cover in which multicellular trichomes form an egg-beater-like structure which is additionally topped by a hydrophilic tip (Barthlott et al. 2010) (Fig. 5b). The surface of the trichomes, as well as the rest of the upper leaf surface, is covered with wax crystals (Fig. 5c). The fascinating interfacial effects caused by this hair cover were studied for several years, because of the anticipated high potential for biomimetic applications, such as drag reducing surfaces for ship hulls (air layers between surface and water decrease drag substantially) and antifouling for immersed objects (the presence of an air layer prevents marine organisms from settling on surfaces) (Barthlott et al. 2017).

Highly promising as the Salvinia Effect is, the benefit of the trichome cover—its effect on fitness—appears to be not really clarified. The leaves are floating, with the lower leaf sides (which is in contact with the water body on which *Salvinia* floats) being not hydrophobic. The upper leaf side, equipped with the hydrophobic trichomes, shows stomata, the typical gas-exchange pores of terrestrial plants (Fig. 5d). When these pores are covered by a water film, photosynthesis will drop because of reduction of diffusional CO_2_ influx. It is arguably beneficial for a floating plant to have a surface which keeps itself reliably free from water, and, therefore, its photosynthesis machinery from being suffocated. However, where is the benefit for a floating plant being equipped with a structure which is able to keep an air layer under water for days? It was suggested that the air layer would serve as a “buoyancy aid”, allowing the plant to rise swiftly to the water surface or to resist being pushed under water (Ditsche et al. 2015). These hypotheses were, however, not tested so far with respect to their selective value for *Salvinia*.

To make things more complex, the different species of *Salvinia* show different kinds of trichomes, from simple solitary trichomes to the complex egg-beater type, which also differ in trichome size and density (Barthlott et al. 2009). All types of trichomes shown by the various *Salvinia* species are superhydrophobic and all are able to keep an air layer for at least some days when immersed in water. *S. oblongifolia*, a species with a less complex trichome type than the complex “egg-beater”, even showed the highest ability for keeping an air layer under water: about 30 days compared to about 13 days in *S. molesta* which features the “egg-beaters” (Ditsche et al. 2015). Therefore, why do different species of *Salvinia* have different trichome types, which differ substantially in their ability to keep air layers under water? What are the selection pressures? Despite the unclear biological function of the “persistent air layer effect” of the *Salvinia* trichomes, this fascinating phenomenon is obviously very promising as a strategy to reduce drag in ships and many other applications (Busch et al. 2019).

As another example, the “bionic car” will be considered. The “bionic car” was devised by Mercedes-Benz as derived from the typical body shape of box fishes (Ostraciidae) (according to Sharfman (2006), the species *Ostracion meleagris* served as original model). Box fishes show—as a group-specific feature—a box-like appearance and a “carapax”, a rigid encasing of the body consisting of a number of fused bony plates which protect the animal from predator attacks. In biomimetic texts describing this innovative car design, a low drag coefficient and an automatic swimming-course stabilization (the latter due to vortices caused by longitudinal keels of the carapax) are attributed to the box-fish body shape, making it a superior biological design example (Allen 2010; Kulfan and Colozza 2012).

Box fishes are reef-dwellers which usually move slowly and are able to maneuver often and skillfully within narrow spaces in their natural habitat, including 180° turns with near-zero turning radii (Van Wassenbergh et al. 2015). When Van Wassenbergh et al. (2015) re-evaluated the hydrodynamic characteristics of the box-fish shape, they could not find any evidence neither for a low drag coefficient nor for a substantial role of stabilizing vortices, in contrast to former studies (Bartol et al. 2005). With regard to the habitat and life style of box fishes, Van Wassenbergh et al. (2015) concluded that a stabilizing system based on automatic vortex production would be of no adaptive value for a fish which must do a lot of maneuvering because the animal would then have to work against the stabilizing vortices, thereby losing energy. It should be added, that there can be also differences in body shape between males and females: for example, in the original model species, *Ostracion meleagris,* only the females have bumps on their heads which are expected to affect flow characteristics (Van Wassenbergh et al. 2015). The results of Van Wassenbergh et al. (2015) were discussed by Webb and Weihs (2015) and Fish and Lauder (2017), with the latter suggesting that the keels might have a stabilizing effect in case of external perturbations, such as irregularly occurring water currents. In short, it appears to be still unclear whether box-fish bodies show adaptations for reduced drag and/or automatic swimming-course-stabilization or not, but nonetheless the bionic car was a successful development which attracted much attention.

The last considered example, the “Petal effect”, describes the combination of high adhesion of a water drop to a surface which is also superhydrophobic, meaning that the sticking drop shows a contact angle of about 150° (Feng et al. 2008). The phenomenon was named after the biological surface for which it was described, the petals of roses (Feng et al. 2008) and has attracted much interest due to its application potential, particularly in microfluidics (Ebert and Bhushan 2012). The Petal effect is mentioned in various biomimetic texts but when originally reported, the authors themselves did not specify a certain biological function of that effect. In fact, it is more than probable that the Petal effect is circumstantially caused by other qualities which have to do with pollination and which result in the “velvet-like” petal surface: optical properties to attract pollinators and a special roughness to facilitate their clinging to and moving on the flower (“Color, gloss and grip”) (Bräuer et al. 2017; Papiorek et al. 2014; Whitney et al. 2009).

## Conclusions: “analog” and “homolog” BID

The examples described so far indicate that there may be no systematic relationship between the proven state of a biological trait effect as an adaptation and the attractiveness and/or success of a BID innovation which was derived from it. When the considered “working principle” is not a working principle but a mere “phenomenon”, it can be nonetheless interesting for technology. In the end—so it appears— the attractiveness of the derived technical application is what counts and not deeper revelations of a complex biological context. Does it then, finally, matter for a BID if the trait effect does not represent an adaptation—or if the technical function is not related to the biological function?

**Fig. 6:**
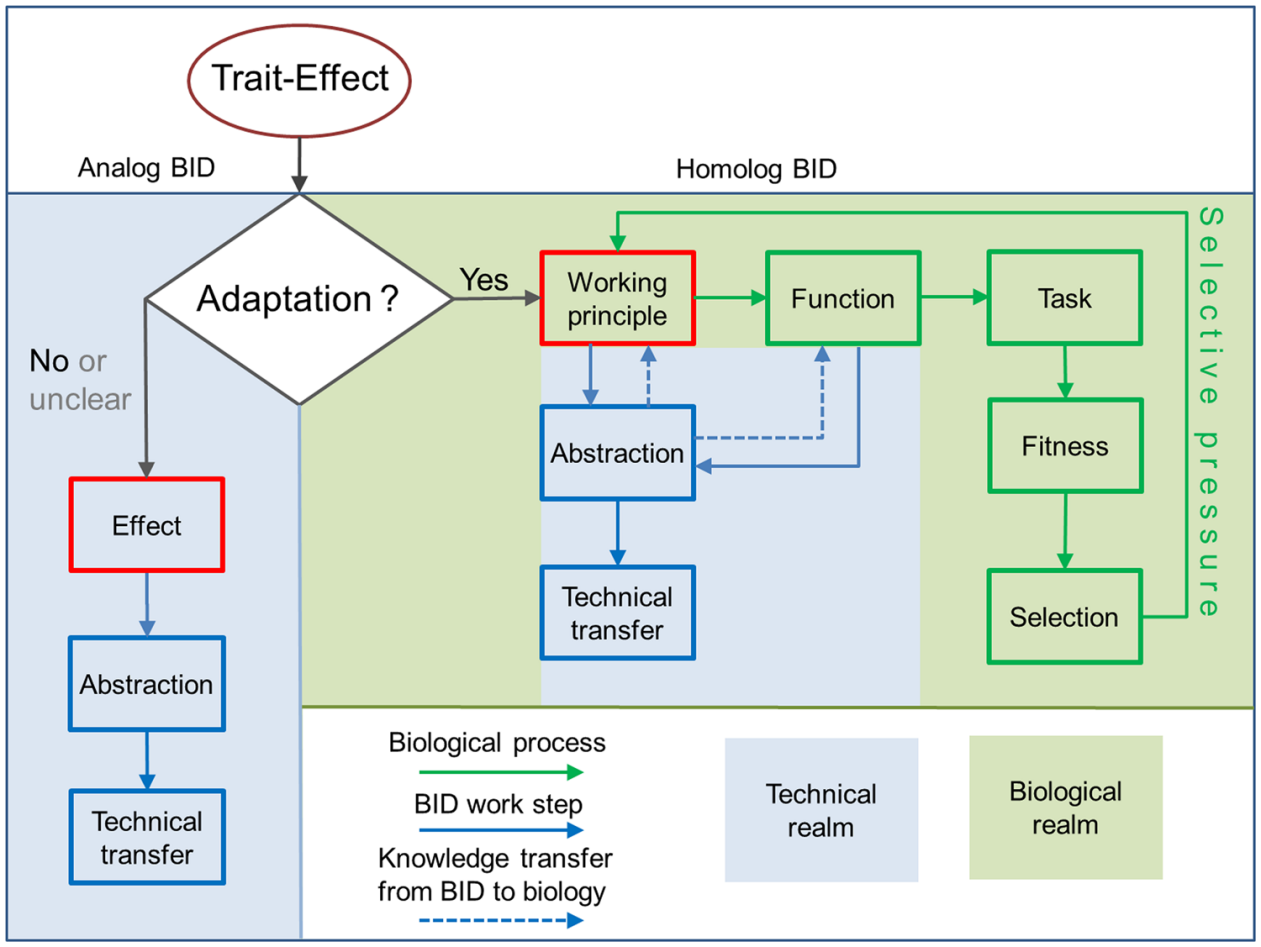
Dichotomy in bio-inspired approaches. If the considered trait is not an adaptation, then the observed trait effect does not represent a working principle but a mere physical phenomenon. Although a successful technical transfer and product is possible, the BID has no further relationships with biology, with consequences for the work program. For example, there will be no “trend of improvement”, because the trait was not shaped by evolution for that effect. In addition, there would be no trade-off problems which are caused by antagonistic functions. If the trait, however, is an adaptation, then the BID project is deeply embedded in biology, and substantial knowledge transfer between biology and BID is to be expected

In fact, it has quite-far reaching consequences. If the trait is not an adaptation—meaning it has no biological functionality contributing to the fitness of the organism—or if the biological function is (yet) unknown, then the abstraction of the observed trait effect does not analyze a working principle but a mere physical phenomenon (Fig. 6). The considered trait effect may well lead to a successful technical transfer and product, but has no further relationships with biology (Fig. 6), with consequences for the research program. For example, there will be no “trend of improvement” which could be further studied and analyzed, because the trait was not shaped by evolution for that effect. This means also that nothing could be learned from comparative analyses which have considerable principal potential for elucidating functional details of biological traits. In addition, there would be no potential trade-off problems which are caused by antagonistic functions. It is suggested to term this kind of BID as “analog BID”.

This is similar to innovations inspired from non-biological effects. For example, a superamphiphobic surface coating based on candle soot was developed based on the observation that candle soot shows remarkable surface effects (Deng et al. 2012). Naturally, the original function of candles was completely irrelevant. Another example is the excellent sound absorption by snow which was studied with respect to technical applicability of materials composed of similarly structured particles (Maysenhölder et al. 2012).

If the trait, however, is an adaptation then the BID project is deeply embedded in biology (Fig. 6). Knowledge on the biological function is then valuable for studying and abstracting the working principle because the analysis can focus on aspects which promote the performance of the biological functionality or are assumed to do so. In addition, biology can then greatly benefit from BID, because new data and information relevant for understanding the organism may be found during a BID project (Speck and Speck 2021). With the same reasoning, it can be evaluated whether and how the biological working principle is constrained by phylogenetic limits and/or trade-off problems or other limitations imposed by various boundary conditions that restrict the degrees of freedom of a biological structure (Broeckhoven and du Plessis 2022). In fact, substantial knowledge transfer between biology and BID is to be expected (Fig. 6). It is suggested to term this kind of BID as “homolog BID”.

For example, the sensing hairs of insects are, on one hand, a model for micro-electromechanical systems (MEMS), due to their outstandingly high performance (Droogendijk et al. 2014). On the other hand, various open questions regarding patterns and length of these hairs with respect to their working principle can be addressed using MEMS as artificial study objects (Casas et al. 2010; Krijnen et al. 2019). In this way, the same technology expected to benefit from the knowledge on sensing hairs can—in turn—contribute to a better understanding of the biological model, opening up possibilities for an improved biomimetic approach (Krijnen et al. 2019). In addition, knowledge about biological traits that goes beyond their essential functions but reveals related aspects may be useful. For example, the superhydrophobic surface of Lotus leaves has a quite limited lifetime, because the leaves have a longevity of about 40 days (Tsuchiya and Nohara 1989), and can, therefore, be viewed as a disposable system.

As a conclusion, research concepts with respect to a BID-related trait and effect will be fundamentally different depending on whether the trait effect is an adaptation or not. Clarifying current knowledge on the adaptive status of a trait and its relationships to the desired technical function should, therefore, be at the beginning of a roadmap for a BID project. It is emphasized at various occasions that incomplete understanding of the considered trait can hamper progress of a BID approach whereas the technical transfer can greatly benefit from the biological context of a trait (Wolff 2017; O´Rourke and Seepersad 2015). Although efforts to reveal adaptive benefits of traits can be quite complex and also sobering, results can be rewarding because they may open up new perspectives and prevent thinking from being “stuck” in preconceived concepts (Bartlett et al. 2012; King et al. 2014; Patek 2014; Wolff et al. 2017). Knowledge on biological traits can also change over time, when new results emerge. To identify adaptations, their functional context, biological limitations and related trade-offs, biologists are clearly needed (Graeff et al. 2020; Snell-Rood 2016).

How deeply, however, must a BID project delve into the biology of its model trait? The quite large amount of biomimetic work which has accumulated so far makes the impression of a multitude of single examples showing many idiosyncrasies with respect to the degree and intensity of interdisciplinary cooperation between biologists and engineers (Speck et al. 2017). In practice, it appears to be quite unresolved which efforts devoted to the biological basics of a trait effect are deemed to be economically justifiable, worthwhile and/or necessary. The biological depth which is finally considered in a BID project seems to be quite arbitrary, depending on project intentions, goals and the degree of interdisciplinary collaboration. It is suggested that at least the rigorous, serious and honest clarification of the adaptive status of a trait effect is an essential and valuable basis for a stringent research concept for a BID project, to allow to distinguish between “analogs” and “homologs”. This kind of “pre-analysis” can have the potential to recognize possible problems and pitfalls as well as to evaluate the real need and benefit of including biological considerations and/or studies in BID-related work.

## Data Availability

Not applicable.
